# Supplementary material for: EnzML: multi-label prediction of enzyme classes using InterPro signatures
Source: BMC Bioinformatics. 2012 Apr 25;13:61. doi: 10.1186/1471-2105-13-61 (PMC3483700; doi:10.1186/1471-2105-13-61)
Supplement: Addtional file 5 — The Java code to format the data files, evaluate and predict. The file enzml_java_code.tar.gz contains the Java code used to format database data to ARFF and XML formats, to execute cross and train-test (jackknife) evaluations and to record evaluation results to database. More information is included in the readme.txt file and the Javadoc files. The code can be used with a MySQL database. To use a different database software, other JDBC drivers might be required. [file 1471-2105-13-61-S5.gz › java_code/enzml2011/doc/uk/ac/ed/inf/taweka/ArffPropsTableReader.html]

ArffPropsTableReader


---


|  |  |  |
| --- | --- | --- |
| **Overview** \| **Package** \| **Class** \| **Use** \| **Tree** \| **Deprecated** \| **Index** \| **Help** | |  |
| **PREV CLASS**   **NEXT CLASS** | **FRAMES**    **NO FRAMES**     **All Classes** |  |
| SUMMARY: NESTED \| FIELD \| CONSTR \| METHOD | DETAIL: FIELD \| CONSTR \| METHOD |  |


---


## uk.ac.ed.inf.taweka Class ArffPropsTableReader

```
java.lang.Object
  uk.ac.ed.inf.utils.database.DbManaged
      uk.ac.ed.inf.utils.database.TableManager
          uk.ac.ed.inf.taweka.ArffPropsTableReader
```

---

``` public class ArffPropsTableReader extends uk.ac.ed.inf.utils.database.TableManager ```

TODO split ArffPropsTableManager in manager/reader/writer/creator

**Version:**
:   14 Jun 2010

**Author:**
:   Luna De Ferrari luna.deferrari-at-ed.ac.uk

---

**Field Summary**

**Fields inherited from class uk.ac.ed.inf.utils.database.TableManager**

`m_writer`


**Constructor Summary**

`ArffPropsTableReader(uk.ac.ed.inf.utils.database.DbManager dbManager)`


| **Method Summary** | |
| --- | --- |
| `java.lang.String` | `getArffPathFrobDb(int recordId)` |
| `java.lang.String` | `getArffPropsPathFrobDb(int recordId)` |
| `java.lang.String` | `getPropertyValueFromTable(java.lang.String propertyName, int id)` |

**Methods inherited from class uk.ac.ed.inf.utils.database.TableManager**

`getTableCreator, getTableName, getTableReader, getTableWriter`

**Methods inherited from class uk.ac.ed.inf.utils.database.DbManaged**

`getDbConnection, getDbManager`

**Methods inherited from class java.lang.Object**

`equals, getClass, hashCode, notify, notifyAll, toString, wait, wait, wait`

**Constructor Detail**

### ArffPropsTableReader

```
public ArffPropsTableReader(uk.ac.ed.inf.utils.database.DbManager dbManager)
```


**Method Detail**

### getArffPathFrobDb

```
public java.lang.String getArffPathFrobDb(int recordId)
```

---


### getArffPropsPathFrobDb

```
public java.lang.String getArffPropsPathFrobDb(int recordId)
```

---


### getPropertyValueFromTable

```
public java.lang.String getPropertyValueFromTable(java.lang.String propertyName,
                                                  int id)
```


---


|  |  |  |
| --- | --- | --- |
| **Overview** \| **Package** \| **Class** \| **Use** \| **Tree** \| **Deprecated** \| **Index** \| **Help** | |  |
| **PREV CLASS**   **NEXT CLASS** | **FRAMES**    **NO FRAMES**     **All Classes** |  |
| SUMMARY: NESTED \| FIELD \| CONSTR \| METHOD | DETAIL: FIELD \| CONSTR \| METHOD |  |


---
